# Correspondence

**Published:** 1882-06

**Authors:** J. E. Janvrin

**Affiliations:** New York


### CORRESPONDENCE.

*Editor of the* INDEPENDENT PRACTITIONER :

Will you kindly call the attention of the readers of your journal to the proposition which I am about to make ?

A "System of Gynæcology by American Authors" is in process of preparation, and it is intended that this shall be as nearly encyclopædic as possible.

To me has been allotted the task of writing the chapter on the "History and Statistics of Ovariotomy." To obtain complete statistics of all ovariotomies done in the three quarters of a century of the history of the operation is a task quite impossible, as many of the earlier ovariotomists are dead, and their records lost. Even among the living many may be disinclined to co-operate with me, as they should. Although the report of each operator's cases may cost him some time and trouble, you will readily see what a valuable fund of information will be obtained by the collation and arrangement of all the facts to be gained.

It is desirable, too, to have all material ready for the press by the end of this year, so that the sooner the returns are made, the lighter will be the task of the editor. Please request all who wish their cases published, to send me the reports before September 1 prox.

May I request your earnest co-operation in the matter, that the work may be worthy the great subject in hand ?

The questions to be answered are as follows :

1. Name of operator ? 2. Age of patient ? 3. Nationality ? 4. Married or single ? 5. Aspiration or previous tapping ? 6. Duration of growth ? 7. Laparotomy or vaginal operation ? 8. Condition of patient at time of operation ? 9. Were antiseptic precautions used ? 10. Was the spray used ? 11. Long or short incision ? 12. Adhesions or other complications ? 13. Double or single ovariotomy ? 14. Pathological features of cyst ? 15. Treatment of the pedicle ? 16. With or without drainage ? 17. Duration of operation ? 18. Complicated or uncomplicated history after operation ? 19. Anti-pyretics used, if any ? 20. Result ? Cause of death, if any ? 21. Primary or secondaryoperation ?

Let the answers be as concise as possible. In many cases a simple yes or no will suffice.

Will you kindly assist in giving the greatest publicity possible to this notice, and publish it for a sufficient length of time in your journal ? Blanks, containing lists of the questions referred to, will be sent to any address.

All communications should be addressed to me at 191 Madison Avenue.

J. E. Janvrin.

New York, May 1, 1882.

